# AA-Amyloidosis Can Be Transferred by Peripheral Blood Monocytes

**DOI:** 10.1371/journal.pone.0003308

**Published:** 2008-10-02

**Authors:** Jana Sponarova, Sofia N. Nyström, Gunilla T. Westermark

**Affiliations:** Division of Cell Biology, Department of Clinical and Experimental Medicine, Faculty of Health Sciences, Linköping University, Linköping, Sweden; Massachusetts Institute of Technology, United States of America

## Abstract

Spongiform encephalopathies have been reported to be transmitted by blood transfusion even prior to the clinical onset. Experimental AA-amyloidosis shows similarities with prion disease and amyloid-containing organ-extracts can prime a recipient for the disease. In this systemic form of amyloidosis N-terminal fragments of the acute-phase reactant apolipoprotein serum amyloid A are the main amyloid protein. Initial amyloid deposits appear in the perifollicular region of the spleen, followed by deposits in the liver. We used the established murine model and induced AA-amyloidosis in NMRI mice by intravenous injections of purified amyloid fibrils (‘amyloid enhancing factor’) combined with inflammatory challenge (silver nitrate subcutaneously). Blood plasma and peripheral blood monocytes were isolated, sonicated and re-injected into new recipients followed by an inflammatory challenge during a three week period. When the animals were sacrificed presence of amyloid was analyzed in spleen sections after Congo red staining. Our result shows that some of the peripheral blood monocytes, isolated from animals with detectable amyloid, contained amyloid-seed that primed for AA-amyloid. The seeding material seems to have been phagocytosed by the cells since the AA-precursor (SAA1) was found not be expressed by the monocytes. Plasma recovered from mice with AA amyloidosis lacked seeding capacity. Amyloid enhancing activity can reside in monocytes recovered from mice with AA-amyloidosis and in a prion-like way trigger amyloid formation in conjunction with an inflammatory disorder. Human AA-amyloidosis resembles the murine form and every individual is expected to be exposed to conditions that initiate production of the acute-phase reactant. The monocyte-transfer mechanism should be eligible for the human disease and we point out blood transfusion as a putative route for transfer of amyloidosis.

## Introduction

Amyloidosis is a heterogeneous group of protein conformational diseases, characterized by accumulation of protein fibrils with distinctive β-pleated structure in different organs and tissues. Hitherto, more than 25 different proteins have been isolated and characterised from amyloid deposits [1]. The mechanisms leading to β-pleated sheet conformation of natural soluble precursor proteins and the propagation of amyloid fibrils are unknown. AA- (reactive) amyloidosis that occurs in patients with rheumatoid arthritis and other chronic inflammatory diseases, results from a sustained elevation of the precursor, apolipoprotein serum amyloid A (SAA). SAA is an acute phase reactant produced mainly by the hepatocytes under regulation by interleukin (IL)-1, IL-6, and tumour necrosis factor [2], [3]. N-terminal fragments (protein AA) of SAA make up the amyloid fibril [4]–[7]. It is a systemic disease and AA amyloid deposits are present throughout the body, but proteinuria is often the first clinical manifestation. AA amyloidosis can be induced experimentally in susceptible mouse strains by inflammatory stimuli that result in a >1,000-fold increase in SAA plasma concentration. Several isoforms of acute phase SAA are known [8], but only SAA1 (former SAA2) serves as a precursor of amyloid fibrils in mice [9].

Development of experimental AA amyloidosis is a biphasic process with a long predeposition phase under which protein aggregates are formed, and a second phase characterized by fibril propagation [10].The time for development of amyloidosis is shortened from several weeks to days in mice after injection of or feeding with extracts from amyloid-laden tissue [11]–[14]. The active component is referred to as ‘amyloid enhancing factor’ (AEF) and has been identified to be the AA fibril itself [13], [15]. In the mouse model, the primary site for amyloid deposition is the spleen followed by deposits in liver, and if the kidneys are engaged this happens at much later time point. However, amyloid at this latter site has been suggested to originate from redistribution of amyloid rather than being recently formed [16].The same distribution pattern is seen both with and without acceleration of amyloidosis by AEF [17]. Rather than being initiated at various sites, this spreading most likely occurs by seeding with preformed amyloid fibrils. How these reach different sites in the body is unknown but theoretically seed can be transferred by blood plasma or by cells. In animal studies, AA-amyloidosis displays great similarity with transmissible spongiform encephalitis (TSE), because both diseases can be induced or accelerated by introduction of aggregates of misfolded proteins and can be transferred between subjects [13], [18]–[22]. It has been shown that prion infectivity can reside in the blood of sheep and humans, and prions were reported to be transmitted by animal blood transfusion even prior to the clinical onset of the disease [23].

Herein, we have analysed if blood cells or plasma recovered from mice with AA-amyloidosis house amyloid-seed that can prime for the disease when transferred to a new animal. We have used the experimental mouse model for AA-amyloidosis and show that monocytes can transfer amyloid-seed but that this is not true for plasma. We also show that the monocytes do not express SAA1, the precursor-protein for AA-amyloid, but they contain intracellular immunoreactivity specific for the protein. The findings indicate that amyloid is phagocytosed by the monocytes and remains intracellularly in a form that retains its seeding activity. In conjunction with inflammation extracellular exposure of this AA-seed triggers development of AA-amyloidosis.

## Results

### Analysis of AEF activity in plasma and peripheral blood monocytes

At first, plasma from amyloidotic mice was analysed for AEF activity. Mice in group A that received AEF and three AgNO_3_ injections developed moderate AA-amyloidosis as quantified from Congo red stained spleen sections. Amyloid did not develop in group B mice that received a single injection of AEF without the subsequent inflammatory stimuli or in untreated group C mice (Table 1). Plasma was recovered from mice in group A–C in which the animals were sacrificed on day 16, and after sonication re-injected into new recipient mice (groups D–F, respectively). These mice received inflammatory stimuli day 1,7,14 and were sacrificed day 16. Spleen sections were stained for amyloid with Congo red and analysed in polarized light. Amyloid was not detected in the spleen of any of the mice in groups D–F. The absence of amyloid in group D mice shows that plasma collected from animals with AA-amyloid does not reside AEF activity and can not trigger development of the disease during chronic inflammation. The absence of amyloid in group D and E mice shows that the amount of AEF given to mice in group A and B is by itself not sufficient for transfer. As expected, mice injected with plasma isolated from untreated animals also lacked seeding activity. Taken together these results show that AA-fibrils don't exist free in circulation and plasma does not serve as a transmitter of AA-amyloid (Table 1).

**Table 1:** Analysis of AEF activity in plasma from mice with AA-amyloidosis.

Treatment of donor mice					Recipient mice	
Group	AEF	AgNO3	No. of mice with amyloid/total no. of mice	Amyloid grade	Group	No. of mice with amyloid/total no. of mice
A	+	+	10/10	2+–3+	D	0/20
B	+	−	0/11	−	E	0/22
C	−	−	0/10	−	F	0/10

Mice from group A received AEF and 0.2 ml 1% silver nitrate on day 1 and further injections of silver nitrate on day 7 and 14, mice in group B received AEF day 1 and mice in group C were untreated. Animals were sacrificed on day 16. The presence of amyloid was analysed in spleen after Congo red staining and plasma was collected, sonicated and 0.1 ml was injected i.v. in new mice (groups D–F). These mice received inflammatory stimuli day 1, 7 and 14 and were sacrificed day 16. The presence of amyloid was analysed in spleen sections after Congo red staining.

**Figure 1:**
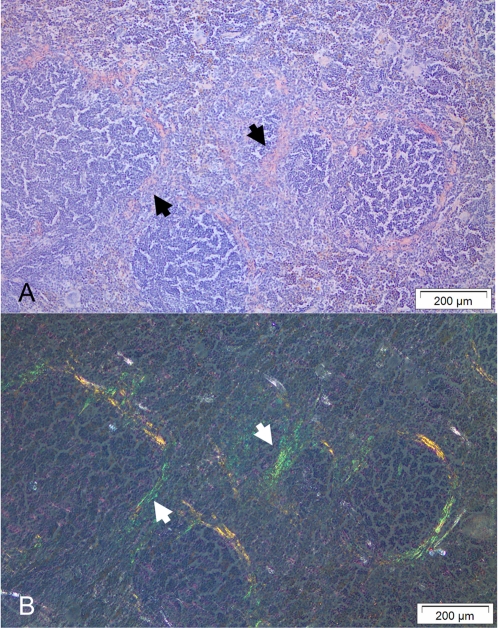
Spleen amyloid deposits stained with Congo red. (A) The amyloid appears pink and is localized to the perifollicular zone. (B) The identical area exhibits green birefringence in polarized light. Amyloid is indicated by arrows.

The study continued with analysis of the possibility that peripheral blood monocytes carry amyloid seeds. Profound amounts of amyloid developed in spleen of group G mice after given AEF and 5 weekly AgNO_3_ injections. Isolated and sonicated peripheral blood monocytes from group G mice were re-injected into new mice (H1-9). Inflammatory stimuli were given to mice in the groups H1-8. These groups varied in size and contained 5–8 animals (Table 2). After Congo red staining, amyloid deposits were detected in 19/48 (40%) of the recipient mice. The amyloid was present in the perifollicular area of the white pulp, and ranged from traces up to moderate amount (1+ to 3+) (Figure 1). The percentage of affected animals differed between groups and in group H2 all mice developed amyloid while this was absent in mice from group H5 and H7 (Table 3). The five animals in group H9 received monocytes isolated from G9 without the concomitant inflammatory stimuli (group H9), and no amyloid developed. Peripheral blood monocytes were also isolated from healthy animals and after sonication injected into nine mice (H10) which received the concomitant inflammatory stimuli. Also in these mice amyloid was absent (Table 2). To validate this phenomenon, peripheral blood monocytes were isolated from the five mice of group H2 that developed AA-amyloid deposits triggered by sonicated peripheral blood monocytes and AgNO_3_ injections. The isolated monocytes were after sonication reinjected into new groups of healthy mice (groups J1–J5) and AgNO_3_ was administered as before. Indeed, after 16 days amyloid was present in 80% (12/15) of the animals in group J 1–5. The degree of amyloid ranged from 1+ to 2+ (Table 4). To study the connection between amyloid load and transmissibility, we tested AEF-activity of monocytes isolated from mice injected with regular AEF and AgNO_3_ and sacrificed at different intervals. Two minutes was the shortest studied time point and this was expected to be sufficient to ensure that AEF has been distributed throughout the body (group K1). Six hours (group K2) were expected to be sufficient for AEF to be phagocytosed by monocytes present in circulation. However, we did not observe any amyloid deposits in mice injected with monocytes isolated from K1 and K2 (groups L1 and L2, respectively), Table 5. In mice sacrificed 48 hrs after AEF and AgNO_3_ injection (group K3) it was possible to detect traces (1+) of amyloid in 3 out of 3 mice, but monocytes isolated from these mice did not reveal any AEF-activity (group L3). The seeding effect was first observed in mice from group L4 that received monocytes isolated from mice sacrificed 7 days after the AEF and AgNO_3_ injection, group K4. Amyloid was detected in 5 out of 9 mice (56%) (Table 5).

**Table 2:** Analysis of AEF activity in peripheral blood monocytes isolated from mice with AA-amyloid induced by AEF.

Treatment of donor mice					Recipient mice		
Mouse	AEF	AgNO3	No. of mice with amyloid/total no. of mice	Amyloid grade	Group	No. of mice with amyloid/total no. of mice	Amyloid grade
G1–G8	+	+	8/8	4+	H1–8	19/48	1+–3+
G9	+	+	1/1	4+	H9	0/5	−
G10	−	−		−	H10	0/9	−

AA-amyloid was induced in nine mice (G1–G9) by an i.v. injection of 0.1 ml AEF with concomitant s.c. injection of 0.2 ml 1% silver nitrate day 1, 7, 14, 21 and 28 and the mice were sacrificed on day 35. The presence of amyloid in the spleen was verified by Congo red staining. Peripheral blood monocytes were isolated, sonicated and re-introduced into the blood circulation of new groups of healthy mice (H1–H8). These mice received inflammatory stimuli day 1, 7 and 14 and were sacrificed day 16. The presence of amyloid was analysed in spleen sections after Congo red staining. Mice in group H9 received sonicated monocytes without subsequent inflammatory stimuli and group H10 received monocytes isolated from untreated mice and subsequent inflammatory stimuli on day 1, 7 and 14 and were sacrificed day 16 (group H10).

**Table 3:** Analysis of AEF activity in peripheral blood monocytes isolated from mice with AA amyloidosis.

Donor mice		Recipient mice		
Mouse	Amyloid grade	Group	No. of mice with amyloid/total no. of mice	Amyloid grade
G1	4+	H1	5/7	1+–2+
G2	4+	H2	5/5	1+–3+
G3	4+	H3	4/8	1+–2+
G4	4+	H4	1/6	1+
G5	4+	H5	0/6	−
G6	4+	H6	2/6	1+–2+
G7	4+	H7	0/5	−
G8	4+	H8	2/5	1+–2+

The table presents detailed information on animals in group H 1–8. AA-amyloidosis was induced by i.v. injection of AEF and 0.2 ml 1% silver nitrate injections on day 1, 7, 14, 21 and 28. The animals were sacrificed on day 35. Blood was collected and amyloid was verified in spleen sections after Congo red staining. Isolated peripheral blood monocytes were injected into new animals, group H 1–8, and silver nitrate was given day 1, 7 and 14.The animals were sacrificed day 16 and the presence of amyloid was studied in spleen after Congo red staining.

**Table 4:** Analysis of AEF activity in peripheral blood monocytes isolated from mice with AA amyloid induced by monocytes isolated from mice with AEF-induced AA amyloid.

Donor mice		Recipient mice		
Mouse	Amyloid grade	Group	No. of mice with amyloid/total no. of mice	Amyloid grade
H2-1	1+	J1	2/3	1+–2+
H2-2	3+	J2	3/3	1+
H2-3	2+	J3	2/3	1+
H2-4	1+	J4	3/3	1+–2+
H2-5	2+	J5	2/3	2+

Mice in group J received an i.v. injection of monocytes isolated from H2 (1–5) and a sequential s.c. injection of 0.2 ml 1% silver nitrate day 1, 7 and 14 and were sacrificed day 16.The presence of amyloid was analyzed in spleen after Congo red staining.

**Table 5:** Analysis of AEF activity in monocytes isolated at different time points after AEF and AgNO_3_ injection.

Treatment of mice prior to monocytes isolation						Recipient mice		
Group	AEF	AgNO3	Duration	No. of mice with amyloid/total no. of mice	Amyloid grade	Group	No. of mice with amyloid/total no. of mice	Amyloid grade
K1	+	+	2 min	0/3	−	L1	0/9	−
K2	+	+	6 hours	0/3	−	L2	0/9	−
K3	+	+	48 hours	3/3	1+	L3	0/9	−
K4	+	+	7 days	3/3	2+	L4	5/9	1+

Mice in group K received an i.v. injection of 0.1 ml AEF with a sequential s.c. injection of 0.2 ml 1% silver nitrate and were sacrificed 2 minutes, 6 and 48 hours, and 7 days later. Peripheral blood monocytes were isolated, sonicated and injected in to new groups with three animals in each, group L. These mice received inflammatory stimuli day 1, 7 and 14 and were sacrificed day 16. The presence of amyloid was analyzed in spleen after Congo red staining.

### Isolation of peripheral blood monocytes

**Figure 2:**
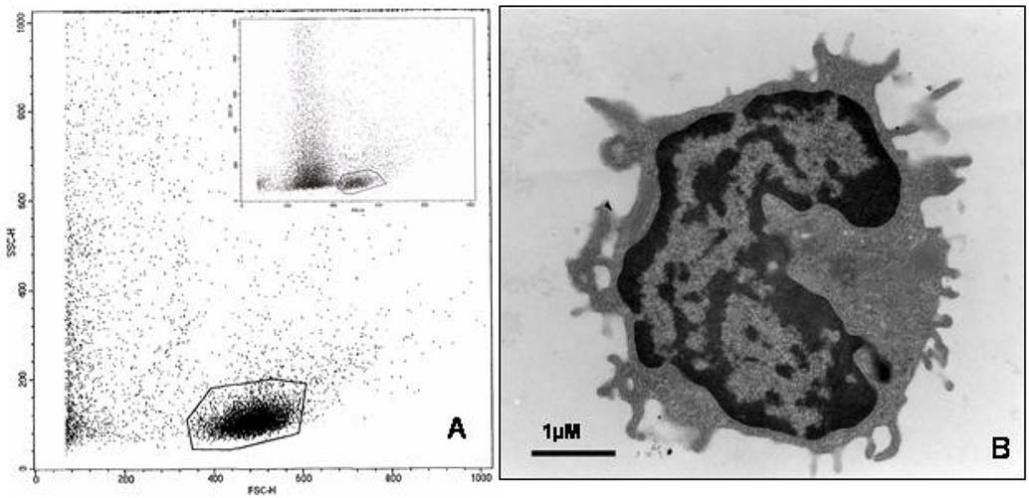
(A) Flow cytometry analysis of isolated and cultured fraction of peripheral blood monocytic cells (PBMC). The measurements were performed using forward scatter versus side scatter and 10,000 events were recorded. The marked area represents monocytic population and four independent isolations were analyzed. The monocyte population was determined to 64%±15% (mean±SD). Insert shows analysis of PBMC prior to culture. (B) Representative picture of a cell recovered after isolation and culture. Bar 1 uM.

Peripheral blood monocytes were isolated from relatively small volumes of 1 ml blood. With the Ficoll-Paque isolation procedure granulocytes are expected to be excluded from the white blood cells that remain on the top of the gradient. During overnight incubation monocytes are expected to adhere to the plastic support while most of the lymphocytes should remain in the non-adherent fraction. Analysis of cells recovered after rinsing and trypsination revealed an enrichment of monocytes. Comparison of cytometry analysis of cell isolations before and after overnight culture shows an enrichment of cells with monocytes appearance in the latter. The monocyte population of the four different isolations varied (64±15%; mean±SD) (Figure 2A). At high resolution it was shown that the majority of isolated cells were monocytes (Figure 2B).

### Detection of AA/SAA reactivity in stimulated monocytes

**Figure 3:**
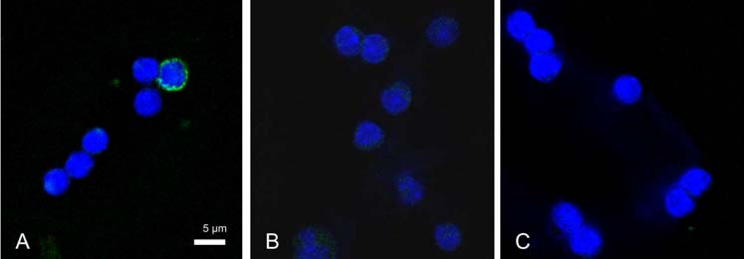
Analysis of AA/SAA reactivity in peripheral blood monocytes by confocal microscopy showed immunoreactivity in 5% of the monocytes isolated from a mouse with AA-amyloidosis (A). There was no reactivity present in monocytes recovered from a mouse given one AgNO_3_ injection 48 hrs prior to isolation (B) or in monocytes isolated from untreated mice (C). The used rabbit antiserum recognizes both protein AA and SAA and was visualized by goat anti rabbit Alexa488-cojugated IgG. Cell nuclei were labeled with TO-PRO3. Bar 10 um.

AgNO_3_ injection generates an acute phase response with striking increase of SAA production within 24 hours. Hepatocytes are the predominant site for production of SAA 1 and SAA2, but extrahepatic production occurs at different sites and SAA 3 is reported to be produced by macrophages and adipocytes. Immunolabeling with antiserum produced against isolated mouse amyloid A, and therefore reactive against SAA 1 and 2, labelled the cytoplasm of 5% of the monocytes isolated from mice with AA-amyloidosis (Figure 3A). No reactivity was present in monocytes isolated from mice that were challenged with one AgNO_3_ injection 48 hours earlier or from monocytes isolated from un-stimulated mice (Figure 3B–C).

### Characterization of SAA expression in peripheral blood monocytes

**Figure 4:**
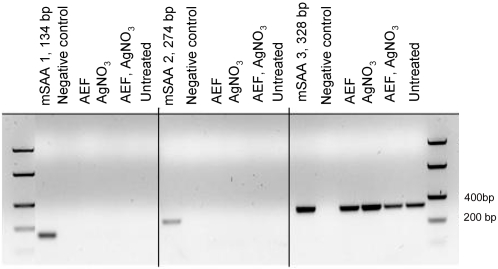
SAA 1, SAA 2 and SAA 3 mRNA expression in peripheral blood monocytes were analysed with PCR. Cells were isolated from mice that developed AA-amyloid after AEF and AgNO_3_ injections or from mice that received AEF or AgNO_3_ injections only or from untreated mice. Expression of the amyloid-prone SAA 1 or non-amyloidogenic SAA 2 was absent in all monocyte preparations. SAA 3 mRNA was detected in all cells independent of treatment. Mouse liver cDNA was used as a positive control. The PCR products were separated on a 1.6% agarose gel.

mRNA was isolated from monocytes from mice that developed AA-amyloidosis after AEF and AgNO_3_-injections, mice that been challenged with either AEF or AgNO_3_ 48 hours prior to isolation and untreated controls. PCR with SAA 3 specific primers amplified a product with the expected size of 328 bp in all four monocyte preparations (Figure 4). The SAA1 and SAA2 specific primers did not amplify any product in any of the monocyte preparations (Figure 4).

## Discussion

We have shown that monocytes isolated from mice with AA amyloid can carry AEF activity and lead to the development of AA amyloidosis in a susceptible recipient. AA-amyloid was detected in the spleen of 19 out of 48 (40%) mice from group H, after injection with monocytes isolated from mice with AA amyloidosis, group G (Table 2 and 3). There was a variability in the degree of transfer, and amyloid did not develop in any mice from group H5 and H7 containing six and five mice, respectively, while amyloid developed in five out of five mice in group H2 (Table 3). We think this large difference between groups most likely relates to technical problems with monocyte preparations and does not reflect variations in seeding efficiency. We used small volumes of blood (1 ml) for isolation and the procedure involves gradient separation, multiple washes and cell culture and at all these steps cells could be lost. Monocytes account only for about 1–3% of the total number of leukocytes in mouse [24] and we only detected AA/SAA reactivity in 5% of the monocyte population (Figure 3A). Hence, if we postulate that the absence of amyloid in H5 and H7 groups results from poor monocyte preparations from G5 and G7 (Table 2 and 3), and only include groups with at least one positive mouse, AA-amyloid was present in 19 out of 37 animals (51%). It was previously shown by our group that AEF can be efficient in a low dose as 15 pg protein [13]. AA-amyloidosis in group G mice was accelerated by AEF injections and it may be argued that injected AEF is transferred from these animals to group H mice. However, the absence of amyloid in mice given plasma recovered from the amyloid laden mice, group D or plasma recovered from mice injected with AEF group E, opposes this possibility. So is also the transfer of seeding activity from monocytes isolated from group H2 mice. In the H2 group, five out of five mice developed AA amyloidosis and monocytes recovered were injected into five new groups, each containing three animals (J1–5). Here AA amyloid appeared in all five groups and affected 80% of the animals (Table 4). This transfer of seeding activity with monocytes isolated from mice in the H groups to mice in the J groups did not involve any AEF injection.

### From where does the AA/SAA reactivity found in the monocytes originate?

The antiserum used in this study was raised against mouse AA, corresponding to residues1–76 of SAA1, and can not differentiate between AA and SAA. Therefore we analysed the mRNA expression of SAA 1, 2 and 3 in isolated cells. Only the non-amyloidogenic SAA 3 mRNA was detected in monocytes, and its expression occurred in all monocyte preparations studied, independent of on ongoing inflammation or presence of amyloid in the mouse (Figure 4). SAA 3 expression has earlier been shown in monocytes/macrophages [25]–[27] so this finding is not surprising. The members of the SAA family resemble each other to a certain degree [8], [28], [29], but in the region 1–76 of SAA 1 and SAA 3 there are 26 amino acid substitutions. This is a major sequence difference and the absence of labelling of monocytes in preparations from mice that received AgNO_3_ alone or untreated mice show that there is no cross reactivity between the used antibody and SAA 3. Instead, the immunolabeling and mRNA expression pattern supports our hypothesis that the monocytes are capable to phagocytose AA amyloid. This amyloid can thereafter remain either intact or partly degraded in the lysosomes. We tried but failed to show an unquestionable full co-localization of AA/SAA reactivity and the lysosome specific marker LAMP-2 [30]. This may partly be due to the narrow cytoplasm of the monocytes, but as visualised by confocal microscopy (figure 3A), the AA/SAA reactivity is present all through the rim-like cytoplasm. These cells were also stained for amyloid with Congo red, but no staining could be detected. This is not unexpected since minute intra lysosomal fibrilar deposits would escape detection, but still be sufficient to exert AEF activity.

### When and where is the amyloid engulfed by the monocyte?

There was a need for a small amount of amyloid to be present in the spleen before isolated monocytes could transfer AEF activity and when animals had been given AEF an silver nitrate, amyloid was not present at any other location at day seven. The site for early amyloid deposition in the spleen is the perifollicular region, an area with direct contact to the marginal zone, occupied by a large number of macrophages. These cells have been implicated in amyloidogenesis based on their close ultrastructural relationship with amyloid deposits [31], [32], phagocytic function [33], and their proteolytic enzymes [34]. This is the region where blood cells can migrate in and out of the circulation and is in contact with the splenic parenchyma, and a putative site where passing peripheral blood monocytes could phagocytize AA-amyloid. It was shown previously that AEF injected into a mouse kept its activity for at least 180 days, and AA amyloid developed to the same extent when AgNO_3_ injections were given [13]. This shows that the AEF is stored in the organism and escapes degradation and clearance. Monocytes reside in the circulation for a limited time and will eventually migrate out to the peripheral organs where they transform into macrophages or related cells [35]. At this location they can stay for many years [36] and therefore may act as reservoirs for AEF.

Transmission of amyloidosis has been shown also for murine apolipoprotein AII (apoAII) amyloidosis [37]. The question can be raised whether also other systemic and localized forms of amyloidosis can be transmissible and whether this is possible in humans. Transmissibility of AA-amyloidosis is not limited to mice but has been demonstrated in hamster [38] and mink [22] and there is no reason to believe that human is a protected species. Iatrogenic transmission of AEF-activity through blood transfusion, similar to what has been shown for variant Creutzfeldt-Jakob's disease [39] can be possible, given that the recipient has a chronic inflammatory disease. It is interesting to note that Brown et al [40] showed prion infectivity of blood from mice clinically ill after inoculation with human transmissible spongiform encephalopathy. Although the degree of transmissibility was low they detected the highest infectivity with the buffy coat while that of plasma and Cohn fractions were lower. This is in agreement with our finding and points to cells as seed carrier and disease primers.

## Materials and Methods

### Animals

Outbreed female 6–8 weeks old NMRI mice (B &K Universal; Södertälje, Sweden), were housed individually and had free access to water and standard chow diet (R70 pellets, Lactamin, Vadstena, Sweden). The study was approved by the Animal Ethics Committee, Linköping University, Sweden.

### Induction of amyloidosis

AEF was extracted from amyloid-laden liver as described earlier [13], and used for amyloid induction in four different groups of mice. Each mouse received 20 µg of protein extract as an intravenous injection in the tail vein. This is further on referred to as AEF-injection. When plasma or peripheral blood monocytes were examined for their ability to transfer AEF activity, sonication 3×20 sec at 23 kHz (MSE Soniprep 150, SANYO, UK) was performed at the time for injection, and a volume of 0.1 ml was injected in the tail vein. The inflammatory challenge was, in all groups, induced by 0.2 ml 1% silver nitrate (AgNO_3_) given as multiple injections subcutaneously.

### Experimental design for plasma analysis

Thirty animals were divided into three groups (A–C). Animals in group A received AEF and 0.2 ml 1% AgNO_3_ on day 1 and additional AgNO_3_ injections on day 7 and 14; animals in group B received AEF only; and animals in group C were untreated. Plasma and spleen were collected when the animals were sacrificed on day 16. Approximately 1 ml of blood was collected from each mouse in heparinized tubes, and plasma was recovered after centrifugation 10 min/2000 rpm/4°C (Allegra X-12R, Beckman, CA, USA). All plasma samples recovered from mice in group A and B were each reinjected into two new animals (group D and E, respectively) (Table 1). Plasma samples recovered from group C were each injected into a single new animal (group F) (Table 1). All these mice (D–F) received 0.2 ml of 1% AgNO_3_ on day 1, 7 and 14 and were sacrificed day 16. The presence of amyloid was investigated in spleen sections after Congo red staining.

### Experimental design for peripheral blood monocyte analysis

Nine mice (group G) received AEF and 0.2 ml 1% AgNO_3_ on day 1 and further AgNO_3_ injections on day 7, 14, 21 and 28. The animals were sacrificed on day 35 and the presence of extensive amyloid was verified in spleen sections after Congo red staining. At the same time blood was collected and monocytes were isolated as described below. These nine isolates were sonicated and their AEF-activity was tested in 9 groups (H1–9) with 5–8 mice in each group (Table 2). In addition to the sonicated monocytes on day 1, animals in groups H1–8 received 0.2 ml of 1% AgNO_3_ on day 1, 7 and 14 while animals in group H9 did not receive any inflammatory challenge. Group H10 received sonicated monocytes isolated from untreated mice and 0.2 ml of 1% AgNO_3_ on day 1, 7 and 14. All animals were sacrificed on day 16. Blood was collected and the presence of amyloid was investigated in sections from spleen after Congo red staining. Group H2 contained five mice which all developed amyloid (Table 3), and monocytes isolated from these were sonicated and injected into five new groups (J1–5) (Table 4), each containing three animals, together with 0.2 ml of 1% AgNO_3_ on day 1, 7 and 14. The presence of amyloid was investigated in sections from spleen after Congo red staining.

To study the connection between amyloid load and transmissibility, twelve animals (groups K1–4) received AEF and a single injection of 0.2 ml of 1% AgNO_3_ (Table 5). Thereafter, animals were sacrificed in groups of three mice at time points 2 minutes (K1), 6 hours (K2), 48 hours (K3) and 7 days post-injection (K4). Blood and spleen was collected when the animals were sacrificed. Monocytes isolated from mice in groups K1–K4 were each injected into three new animals (36 mice), groups L1–L4. These animals were also given 0.2 ml of 1% AgNO_3_ day 1, 7 and 14 and sacrificed on day 16. Spleen was recovered and the presence of amyloid was analysed in sections after Congo red staining.

### Isolation of peripheral blood monocytes

Approximately 1 ml of blood was collected from each mouse in heparinized tubes, diluted 1∶1 with 0.9% NaCl solution and overlaid (1∶1) on Ficoll-Paque™ Premium gradient (GE Healthcare Bio-Sciences AB, Uppsala, Sweden) and centrifuged 40 min/400×g/16°C (Allegra X-12R centrifuge). Peripheral blood mononuclear cells were recovered and remaining erythrocytes were lysed with ice cold water. Cells were incubated in RPMI-1640 medium (Sigma Aldrich, Stockholm, Sweden) supplemented with 10% fetal calf serum at 37°C in an atmosphere of 5% CO_2_ over night. Non-adherent cells were rinsed of and adherent cells were released by trypsination and pelleted (10 min/100×g). Cells analysed for AEF-activity were resuspended in 3 ml of sterile water and stored at 4°C, until used.

### Flow cytometry

Isolated cells from untreated mice were fixed in 4% paraformaldehyde (PFA), pH 7.4 for 15 min at 4°C and resuspended in PBS. Flow cytometry was carried out on four independent isolations (FACS Calibur, Becton Dickinson, San Jose, CA, USA). Monocytes were identified based on their forward scatter versus side scatter, and 10,000 events were recorded.

### Histology

Spleen was fixed in 10% neutral buffered formalin and embedded in paraffin. The presence of amyloid was investigated in 10 um thick sections after Congo red staining [41] and the amyloid amount was quantified according to the following scale: 0; absent; 1+ trace of amyloid; 2+ small amyloid deposits; 3+ moderate amyloid deposits; 4+ extensive amounts of amyloid [13].

### Confocal microscopy

Isolated monocytes were fixed in 4% PFA, pH 7.4 for 20 min at 4°C and used for cytospin preparation by centrifugation (3 min, 500 rpm; Cytospin3 cytocentrifuge, Shandon, UK). Cells were incubated with antibodies raised against mouse amyloid protein A at a dilution 1∶200 over night, at 4°C. This in house produced rabbit antiserum recognizes both AA and SAA. The immunoreactivity was visualized with goat anti-rabbit IgG – Alexa Fluor 488 (Molecular Probes, Eugene, OR, USA) diluted 1∶1000, 2 hrs at room temperature. Incubations and washing steps were performed in the presence of 0.1% Saponine in BSS buffer (137 mmol/l NaCl, 5.36 mmol/l KCl, 1.26 mmol/l CaCl_2_, 811 µmol/l MgSO_4_, 441 µmol/l KH_2_PO_4_, 1.4 mmol/l Na_2_HPO_4_). Slides were mounted with glycerol/PBS (1∶1) in the presence of nuclear stain TO-PRO-3 (Molecular Probes). Cells were examined with a Nikon eclipse E600 microscope connected to a Nikon C1 confocal unit with argon 488 and HeNe 543 lasers (Nikon, Kawasaki, Japan). Digital pictures were taken with an EZ-C1 digital camera and software version 1.0 for Nikon C1 confocal microscopy.

### Electron microscopy

Cells were fixed in 2% PFA with 0.25% glutaraldehyde in PBS for 1 h, post-fixed in 1% OsO_4_ and embedded in Epon (Ladd Research Industries, Burlington, VT, USA). The material was studied at 100 kV in a Jeol 1230 electron microscope (Jeol, Akishima, Tokyo, Japan). Electron micrographs were taken with a Gatan multiscan camera model 791 with Gatan digital micrograph software version 3.6.4 (Gatan, Pleasanton, CA, USA).

### Characterization of SAA expression in peripheral blood monocytes

mRNA was isolated from peripheral blood monocytes (QuickPrep™ *Micro* mRNA purification kit, GE Healthcare, Sweden) and 20 µl of the reaction mixture was used for first-strand DNA synthesis (First-Strand cDNA Synthesis kit, Amersham Biosciences, Sweden). SAA1-3 expression was analysed by PCR with the following primer sequences: SAA 1: forward primer 5′GAAGCTGGCTGGAAAGATG and reverse primer 5′GCTTTCAGGAATTCTTCGG (NM_011314); SAA 2: forward primer 5′GGAGGGTTTTTTTCATTTGTTC and reverse primer 5′TGCTGACCAGGAAGCCAA (NM_009117) and SAA3: forward primer 5′GCCTTCCATTGCCATCATT and reverse primer 5′AGTGGCAAAGACCCCAAC (BC055885). The PCR was performed with 5 µl of the first-strand DNA preparation and 10 µmol/l of each primer under the conditions: denaturation 95°C for 30 sec, annealing 45°C for 30 sec, and elongation 72°C for 1 min for 33 cycles. Mouse liver cDNA was used as positive control. The products were analyzed on 1.6% agarose gel and visualized with Gel Doc™ XR gel documentation system (Bio-Rad, Milan, Italy).
